# Current rice production is highly vulnerable to insect-borne viral diseases

**DOI:** 10.1093/nsr/nwac131

**Published:** 2022-07-06

**Authors:** Jian-guo Wu, Guo-yi Yang, Shan-shan Zhao, Shuai Zhang, Bi-xia Qin, Yong-sheng Zhu, Hui-ting Xie, Qing Chang, Lu Wang, Jie Hu, Chao Zhang, Bao-gang Zhang, Da-li Zeng, Jian-fu Zhang, Xian-bo Huang, Qian Qian, Shou-wei Ding, Yi Li

**Affiliations:** Vector-borne Virus Research Center, Fujian Province Key Laboratory of Plant Virology, Institute of Plant Virology, Fujian Agriculture and Forestry University, China; Vector-borne Virus Research Center, Fujian Province Key Laboratory of Plant Virology, Institute of Plant Virology, Fujian Agriculture and Forestry University, China; Vector-borne Virus Research Center, Fujian Province Key Laboratory of Plant Virology, Institute of Plant Virology, Fujian Agriculture and Forestry University, China; Vector-borne Virus Research Center, Fujian Province Key Laboratory of Plant Virology, Institute of Plant Virology, Fujian Agriculture and Forestry University, China; Institute of Plant Protection, Guangxi Academy of Agricultural Sciences, China; Rice Research Institute, Fujian Academy of Agricultural Sciences, China; Vector-borne Virus Research Center, Fujian Province Key Laboratory of Plant Virology, Institute of Plant Virology, Fujian Agriculture and Forestry University, China; Vector-borne Virus Research Center, Fujian Province Key Laboratory of Plant Virology, Institute of Plant Virology, Fujian Agriculture and Forestry University, China; Vector-borne Virus Research Center, Fujian Province Key Laboratory of Plant Virology, Institute of Plant Virology, Fujian Agriculture and Forestry University, China; Vector-borne Virus Research Center, Fujian Province Key Laboratory of Plant Virology, Institute of Plant Virology, Fujian Agriculture and Forestry University, China; Vector-borne Virus Research Center, Fujian Province Key Laboratory of Plant Virology, Institute of Plant Virology, Fujian Agriculture and Forestry University, China; Vector-borne Virus Research Center, Fujian Province Key Laboratory of Plant Virology, Institute of Plant Virology, Fujian Agriculture and Forestry University, China; State Key Laboratory of Rice Biology, China National Rice Research Institute, Chinese Academy of Agricultural Sciences, China; Rice Research Institute, Fujian Academy of Agricultural Sciences, China; Rice Research Institute, Sanming Academy of Agricultural Sciences, China; State Key Laboratory of Rice Biology, China National Rice Research Institute, Chinese Academy of Agricultural Sciences, China; Department of Microbiology and Plant Pathology, Center for Plant Cell Biology, Institute for Integrative Genome Biology, University of California, Riverside, USA; The State Key Laboratory of Protein and Plant Gene Research, School of Life Sciences, Peking University, China

Rice is one of the most important food crops and feeds more than half of the world’s population [1]. Arthropod-borne rice viruses have caused devastating epidemics in Asian countries and are a major threat to food security [2]. However, little is known about the vulnerability of rice crops to viral pathogens [3,4], especially southern rice black-streaked dwarf virus (SRBSDV), rice black-streaked dwarf virus (RBSDV) and rice gall dwarf virus (RGDV), known to induce annual outbreaks by insect transmission in some localities in Asia.

To address this question, we first investigated the susceptibility of 136 conventional and hybrid rice varieties widely cultivated or approved for release in China to SRBSDV, which has been circulating in Asian countries since its first report in 2008 [2]. We monitored symptom development and asymptomatic infection in seedlings after inoculation with viruliferous white-backed plant hoppers (*Sogatella furcifera*, Horváth) in a greenhouse. We found that most of the varieties examined were highly susceptible to SRBSDV and developed the characteristic disease symptoms at 45 days post-inoculation (Fig. 1A and Supplementary Table S1).

**Figure 1.**
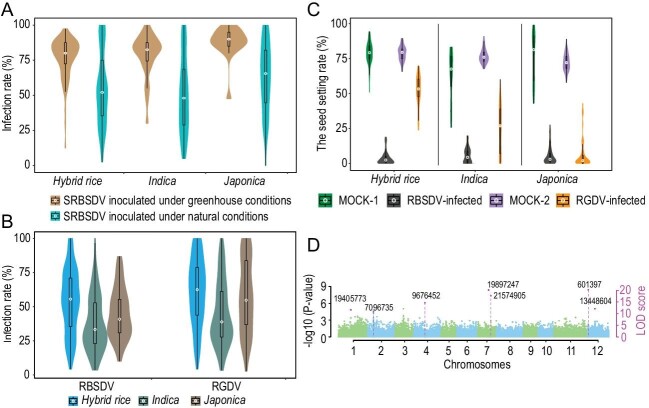
Infection rate under natural and greenhouse conditions and statistical disease indicators comparison of rice varieties upon RBSDV and RGDV infection and the seven identified loci through GWAS analysis. (A) The infection rate of hybrid rice, *indica* type and *japonica* type upon SRBSDV infection under greenhouse and natural conditions. (B) The infection rate of 528 rice varieties upon RBSDV and RGDV infection under natural conditions. (C) Comparison of the seed setting rate (SSR) of 20 rice varieties between mock and RBSDV or RGDV infection, respectively. (D) The seven loci associated with rice tolerance to viral infections identified from Manhattan plots in indicate rice varieties. The left *y*-axis reports –log10 *P*-values, which are obtained from single-marker genome-wide scanning for all the markers in the first step of 3VmrMLM, and the right *y*-axis reports LOD scores, which are obtained from a likelihood ratio test for significant and suggested QTNs, with the threshold of LOD = 10.0 (gray dashed line), in the second step of 3VmrMLM described in Li *et al.* (2022). The LOD scores, along with their quantitative trait nucleotides (QTNs), are shown in points with straight lines. All the main-effect QTNs (red dots) are identified. The number on the red dot represents the chromosomal physical location of the peak of QTN, corresponding to the reference *Nipponbare*.

The results from greenhouse inoculation with viruliferous insect vectors predict widespread vulnerability of rice cultivars to SRBSDV in rice fields. To test this hypothesis, we planted seedlings of

528 varieties in 3 consecutive years under open field conditions at locations of Nanning and Guilin, Guangxi Province that have recorded a multiyear SRBSDV outbreak from plant hopper transmission. We found that ≥25% of the seedlings from 80%–93% of the examined hybrid or conventional *indica* and *japonica* cultivars became infected naturally with SRBSDV 60 days after seedling transplantation (Fig. 1A and Supplementary Table S2).

RBSDV and RGDV outbreaks occur annually in Kaifeng and Yunxiao of Henan and Fujian provinces, respectively. Our field evaluation of the 528 varieties at the two locations from 2016 to 2021 further revealed widespread vulnerability of these rice cultivars to both RBSDV and RGDV, which are transmitted by small brown plant hoppers (*Laodelphax striatellus*, Fallén) and zigzag leaf hoppers (*Deltocephalus dorsalis*, Motschulsky), respectively (Fig. 1B and Supplementary Table S2). A total rice category of infection rate was conducted based on 528 varieties, including different rice types of *indica*, *japonica*, hybrid *indica*, hybrid *japonica*, restorer line, sterile line and maintainer line that were infected by SRBSDV, RBSDV and RGDV, respectively (Supplementary Fig. S1 and Supplementary Table S2). These results indicate that rice varieties currently in production and promotion generally lack broad-spectrum resistance to rice viruses.

To assess the impact of viral infection, 10 seedlings from each of 20 varieties randomly selected either uninfected or infected with RBSDV or RGDV were grown to maturity for calculating seed setting rate (SSR). We found that infection with either RBSDV or RGDV drastically decreased the SSR of both conventional and hybrid cultivars (Fig. 1C and Supplementary Table S3). Together, our findings reveal that rice production and food security are dangerously vulnerable to potential epidemics caused by any of the three insect-borne viral pathogens.

We further used a recently developed methodology framework (3VmrMLM) of genome-wide association studies (GWAS) to search for QTNs (quantitative trait nucleotides) loci associated with viral tolerance based on the data sets from Nanning and Guilin and the available genomic sequences of rice varieties [5–8]. Seven QTNs located on chromosomes 1, 2, 4, 7 and 12 were found to be significantly associated with viral tolerance (Fig. 1D and Supplementary Table S4). Thus, it seems possible to investigate the genetic mechanisms of rice viral resistance using the experimental system established in this work as shown recently in Arabidopsis [9].

Major advances have been made in understanding the mechanisms of important breeding traits of rice such as high yield and rice blast disease resistance [10–15]. Current rice-breeding programs demand resistance evaluation against major fungal and bacterial pathogens. However, the direct link between the agro-ecosystem changes and the outbreaks of rice virus diseases needs attention. Our study shows that the rice varieties popularized in production generally lack antiviral function under both natural and greenhouse conditions and viral infection significantly reduce rice production. Based on our findings, we conclude that there is an urgency to develop a standardized protocol for assessing the performance of current and future rice varieties against key insect-borne viral pathogens to be incorporated into variety certification and approval guidelines.

## Supplementary Material

nwac131_Supplemental_Files
