# Supplementary material for: Current rice production is highly vulnerable to insect-borne viral diseases
Source: Natl Sci Rev. 2022 Jul 6;9(9):nwac131. doi: 10.1093/nsr/nwac131 (PMC9511884; doi:10.1093/nsr/nwac131)
Supplement: nwac131_Supplemental_Files [file nwac131_supplemental_files.zip › Supplemental_Meterials_and_figure_legends.docx]

**Supplementary Materials**

**Title**

**Current rice production is highly vulnerable to insect-borne viral diseases**

**Authors**

Jian-guo Wu^1*^, Guo-yi Yang^1†^, Shan-shan Zhao^1†^, Shuai Zhang^1†^, Bi-xia Qin^2†^, Yong-sheng Zhu^3†^, Hui-ting Xie^1^, Qing Chang^1^, Lu Wang^1^, Jie Hu^1^, Chao Zhang^1^, Bao-gang Zhang^1^, Da-li Zeng^4^, Jian-fu Zhang^3^, Xian-bo Huang^5^, Qian Qian^4*^, Shou-wei Ding^5*^, Yi Li^6*^

*1 Vector-borne Virus Research Center, State Key Laboratory of Ecological Pest Control for Fujian and Taiwan Crops, Institute of Plant Virology, College of Plant Protection, Fujian Agriculture and Forestry University, Fuzhou, 350002, China.*

*2 Institute of Plant Protection, Guangxi Academy of Agricultural Sciences, Nanning, 530007, China.*

*3 Rice Research Institute, Fujian Academy of Agricultural Sciences, Fuzhou, 350018, China.*

*4 State Key Laboratory of Rice Biology, China National Rice Research Institute, Chinese Academy of Agricultural Sciences, Hangzhou,10006, China*

*5 Rice Research Institute, Sanming Academy of Agricultural Sciences, Sanming, 365509, China*

*6 Department of Microbiology and Plant Pathology, Center for Plant Cell Biology, Institute for Integrative Genome Biology, University of California, Riverside, CA, USA.*

*7 The State Key Laboratory of Protein and Plant Gene Research, School of Life Sciences, Peking University, Beijing, 100871, China.*

*^†^ These authors contributed equally to this work.*

**For correspondence:* [wujianguo@fafu.edu.cn; qianqian188@hotmail.com](mailto:wujianguo@fafu.edu.cn;%20qianqian188@hotmail.com); [shou-wei.ding@ucr.edu](mailto:shou-wei.ding@ucr.edu); [liyi@pku.edu.cn](mailto:liyi@pku.edu.cn)

**Materials and methods**

**Natural inoculation**

For examining the performance of different rice germplasms under natural field conditions, we chose four sites that located in Nanning and Guilin, Guangxi Province, China; Kaifeng, Henan Province, China and Yunxiao, Fujian Province, China. Among them, Nanning and Guilin are for testing SRBSDV, Kaifeng is for testing RBSDV and Yunxiao is for testing RGDV. In these four sites, rice viral disease takes place almost every year and infection rates are much higher than other rice growing areas. More than 30 seedings with 40-day-old (8-9 leaves) per rice germplasm were planted in sites of the experimental field with an interval of 15 cm and a row spacing of 20 cm. No pesticides and insecticides were used during the period. The rice germplasms were inspected for rice virus symptoms at 60 days after transplanting. The main symptoms of SRBSDV and RBSDV-infected rice plants were stunting, leaf roll and darkening leaves with wrinkles; the main symptoms of RGDV-infected rice plants were dwarf, curly leaf tip and tumor like protrusion on the back of leaf. We judged plants with an obvious disease phenotype as symptomatic infected rice, and those without disease phenotype but carrying virus using RT-qPCR as asymptomatic infected rice. The variety TN1 was used as a positive control.

**Greenhouse inoculation**

To acquire viruliferous WBPH (white-backed plant hoppers) population with high virus carried rate and overcome the limited life span of WBPH, we allow 20 female WBPH adults that finished in mating feed on SRBSDV-diseased rice plants, and 3-5 days later, the female WBPH adults lay eggs on SRBSDV-infected rice plants. About 7 days later, nymphs begin to hatch, then feed on SRBSDV-infected rice and acquire virus. About 10-12 days later after a 9 days latent period, the nymphs will be used for virus inoculation.

In greenhouse experiment, 40 seedings per rice germplasm were planted in glasshouse conditions at 28°C and a photoperiod of 16 hours of light and 8 hours of dark. The 15-day-old rice seedlings were transferred to the inoculation room for viruliferous WBPH inoculation. The temperature was 25 ℃, and the photoperiod was 16 hours light and 8 hours dark. The seedlings inoculated for 3 days were transferred to a greenhouse for cultivation and statistical analysis was conducted 45 days post inoculation. We judged plants with an obvious disease phenotype as symptomatic infected rice, and those without disease phenotype but carrying virus using RT-qPCR as asymptomatic infected rice.

**RT-qPCR amplification**

Total RNA was extracted from rice plants with Trizol (Life Technologies) following the manufacturer’s instructions, the concentration of total RNA was determined using a NanoDrop 2000 spectrometer (Thermo Fisher Scientific) and integrity was evaluated elec-trophoretically in an agarose gel by visualizing under ultraviolet light. 1 μg of total RNA was reverse transcribed into cDNA by cDNA Synthesis SuperMix (Novoprotein). RT-qPCR amplification was performed in 20 μL reactions containing 4 μL of 10-fold diluted cDNA, 0.5 mM of each primer, and 10 μL of 2 × RealStar Green Fast Mixture (GenStar). Primers SRBSDV-S10-F (5’-TTGACATAGCGCCCGATCTT-3’) and SRBSDV-S10-R (5’- ACGCGATGTGGGGTGATTTA-3’) with expected length of 150 bp, RBSDV-S10-F (5’-GAGCGCAGTAGCGTCATTTG-3’) and RBSDV-S10-R (5’- GAAGCAGAGGTGCCATCGTA -3’) with expected length of 126 bp, RGDV-S8-F (5’- GGCGTCAGTGGGATTTCTGA-3’) and RBSDV-S8-R (5’- ACGTTTGGCGCTACATCTGA-3’) with expected length of 141 bp were used to amplify the S10 genomic segment of SRBSDV, S10 genomic segment of RBSDV and S8 genomic segment of RGDV. Amplified products were run on 2% agarose gel to confirm the specificity of amplification. The TN1 variety cDNA sample containing virus was diluted gradient (from 10^-1^ to 10^-6^), and the cDNA without virus was used as the negative control to verify the specificity of primers and determine the Ct value of virus content limit.

**Data analysis**

Plants with obvious disease phenotype as symptomatic infected rice, and without disease phenotype but carrying virus through RT-qPCR confirmation as asymptomatic infected rice. The infection rate was calculated by dividing the sum of symptomatic infected rice and asymptomatic infected rice with total plants. According to the infection rate, Level 0=0%，Level 1=1–10%，Level 3=11–30%，Level 5=31–50%，Level 7=51–70%，Level 9=71–100%. Level 9 is recorded as highly susceptible variety (HS)。

10 seedlings from each of 20 varieties randomly selected either uninfected or infected with RBSDV or RGDV were harvested, in which the virus was determined by the detection of CP gene through conducting RT-qPCR assays. The seed setting rate (SSR) per plant was calculated through dividing the total number of FGPP (filled grains per panicle) per plant by the total number of GPP (grains per panicle) per plant.

**GWAS for detecting QTNs of rice antiviral**

The indicated rice varieties were genotyped by 157,225 SNP markers. They were phenotyped by incidence of SRBSDV-infected rice. A new methodological framework, 3VmrMLM was used to detect all types of loci and estimates their effects. 3VmrMLM is a three-variance component mixed model was integrated with our multi-locus random-SNP-effect mixed linear model (mrMLM) method^1^. To balance the high power and low false-positive rate for associated SNP detection, a likelihood of odd score of 10.0 was used as a cutoff in the multilocus GWAS.

**Supplemental Figure and Figure Legends**

**
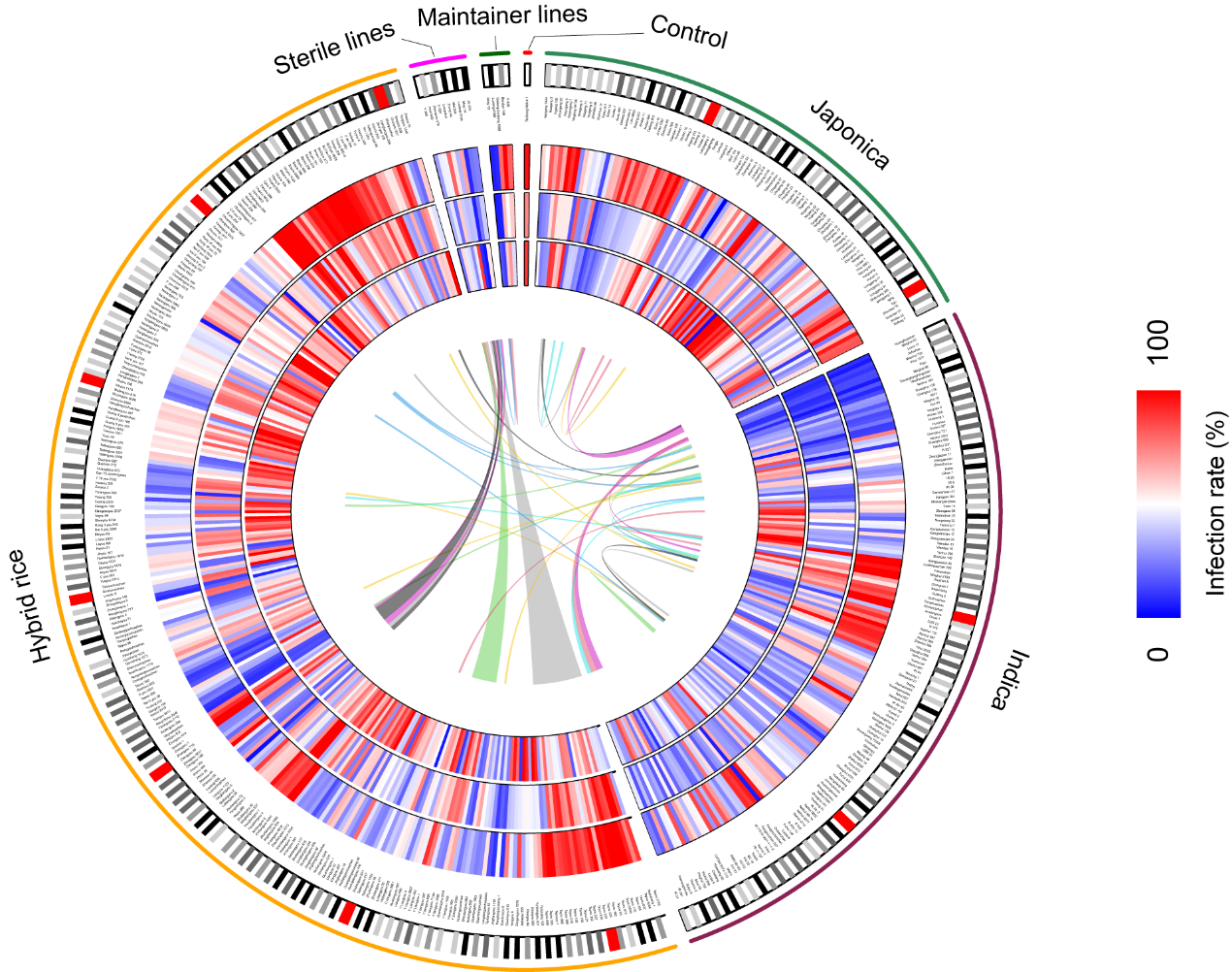
**

**Supplemental Figure 1. Rice category of infection rate**

Rice category of infection rate in the field. The circles (from outermost to innermost) represent rice variety, disease incidence of SRBSDV, RBSDV and RGDV, respectively, and the relationship between offspring and the male parent or female parent. 528 rice germplasms were categorized into restorer lines, hybrid rice, sterile lines and maintainer lines. The variety TN1 was used as a positive control.

**Table S1.** **Statistical data of 136 rice varieties inoculated with SRBSDV under greenhouse conditions**

136 rice germplasms were infected with SRBSDV at 15-day-old stage. The infection rate of rice was assessed at 45 days post inoculation. The variety TN1 was used as a positive control.

**Table S2. Statistical data of 528 rice varieties inoculated with SRBSDV, RBSDV and RGDV under natural conditions, respectively**

For assessed the resistance of rice, 528 rice germplasms including the previous 136 rice varieties were infected by SRBSDV, RBSDV and RGDV under natural conditions. We selected four natural disease areas in Nanning and Guilin, Guangxi Province, China; Kaifeng, Henan Province, China and Yunxiao, Fujian Province, China, as SRBSDV, RBSDV and RGDV experimental sites, respectively. More than 30 seedings with 40-day-old （8-9 leaves）per rice germplasm were planted in sites of the experimental field. The rice germplasms were inspected for rice virus symptoms at 60 days after transplanting. The variety TN1 was used as a positive control.

We judged plants with an obvious disease phenotype as symptomatic infected rice, and those without a disease phenotype but carrying virus as asymptomatic infected rice. The infection rate was calculated by dividing the sum of symptomatic infected rice and asymptomatic infected rice with total plants.

**Table S3. Statistical data of SSR**

10 seedlings from each of 20 varieties of virus-inoculated rice varieties were analyzed the effect of virus infection on yield loss per individual plant through comparison of statistical indicators including number of effective panicles per plant (EPP), grains per panicle (GPP) and filled grains per panicle (FGPP). The seed setting rate (SSR) per plant was calculated through dividing the total number of FGPP per plant by the total number of GPP per plant.

**Table S4. Seven QTNs related to viral tolerance identified by GWAS in rice**

Seven QTNs and the locus ID and putative function of the seven genes were identified according to GWAS, and they were located on chromosomes 1, 2, 4, 7, 12. The positions were indicated in the Table S4.

**Reference**

1 Li, M. *et al.* A compressed variance component mixed model for detecting QTNs and QTN-by-environment and QTN-by-QTN interactions in genome-wide association studies. *Molecular plant* **15**, 630-650, doi:10.1016/j.molp.2022.02.012 (2022).
